# How does the snakehead *Channa argus* survive in air? The combined roles of the suprabranchial chamber and physiological regulations during aerial respiration

**DOI:** 10.1242/bio.029223

**Published:** 2018-01-22

**Authors:** Ting Duan, Chenchen Shi, Jing Zhou, Xiao Lv, Yongli Li, Yiping Luo

**Affiliations:** 1 Key Laboratory of Freshwater Fish Reproduction and Development, Ministry of Education, School of Life Sciences, Southwest University, Chongqing 400715, China; 2 Department of Clinical Medicine, Chongqing Medical and Pharmaceutical College, Chongqing 401331, China

**Keywords:** Respiration, *Channa argus*, Metabolic demand, Oxygen consumption, Anaerobic metabolism

## Abstract

This study aimed to test the hypothesis that the aerial survival of the northern snakehead is involved not only with suprabranchial chamber respiration but also with physiological regulations. The aerial survival time and oxygen consumption rate (VO_2_) were determined in snakeheads with either normal or injured suprabranchial organs. Some hematological and biochemical parameters were assessed during aerial exposure. The results showed that resting VO_2_ decreased when switching from water to air in both the control and the suprabranchial organ-injured fish, with decreases of 22.4% and 23.5%, respectively. Resting VO_2_ in air was not different between the control and the suprabranchial organ-injured fish. The red blood cell (RBC) count and hemoglobin concentration showed no marked changes, while RBC size increased when exposed to air. The liver lactate concentration remained unchanged, and the white muscle lactate concentration decreased when switching from water to air. The blood ammonia concentration tended to increase during aerial respiration. These results suggest that the aerial survival of the snakehead is positively associated with a combination of factors, including respiration of suprabranchial organs and other accessory organs, depressed metabolic demands and increased oxygen transport, and negatively associated with the accumulation of blood ammonia but not anaerobic metabolism.

## Abstract (precis)

**Summary:** The aerial survival of the northern snakehead could be involved with suprabranchial chamber respiration, and also with physiological regulations.

## INTRODUCTION

The northern snakehead (*Channa argus*) is a species of air-breathing fish that is widely distributed in East Asia. This fish has a peculiar accessory breathing organ, the suprabranchial chamber, by which the fish breathes air at the water's surface using a cough-like mechanism (Ishimatsu and Itazawa, 1981; Lefevre et al., 2014), making possible short-term survival out of water (Nagata and Nakata, 1988). The suprabranchial chamber of *Channa* possesses a richly vascularized wall for gas exchange. It communicates with the buccopharyngeal cavity through a ventral opening guarded by a ‘shutter’ plate bone outgrowth of the first branchial arch, which can close the inhalant aperture of the suprabranchial chamber during expiration (Munshi, 1962). Even though many air-breathing fish maintain equivalent oxygen consumption in air and in water (Yoshiyama and Cech, 1994; Sayer, 2005), some air-breathing fish reduce their oxygen consumption rate (VO_2_) in air (Garey, 1962; Martin et al., 2004; Lefevre et al., 2014). Our recent study has found that the northern snakehead can depress metabolic level in air (Li et al., 2017), implying that the snakehead may have mechanisms other than its suprabranchial chamber to meet metabolic demands and survive out of water.

In addition to ventilation, some other physiological regulations may be necessary for fish to breathe air (Sayer, 2005). Fish may improve their blood oxygen-carrying capacity to compensate for their impaired oxygen uptake and/or enhance anaerobic metabolism (which is indicated by lactic acid concentration) during aerial exposure (Farmer, 1979; Bennett, 1978; Graham, 1997). Whether these regulations occur in the northern snakehead during aerial exposure to compensate for air respiration of the suprabranchial organ remains unclear.

Short-term aerial survival has been reported in several *Channa* species, e.g., 8 h for *C. striata*, >27 h for *C. batrachus* (S. Chandra, Histopathology of respiratory organs of fishes, PhD thesis, Banaras Hindu University, 2001; Chandra and Banerjee, 2004), and 14-24 h for the northern snakehead, depending on ambient temperature and metabolic demand (Li et al., 2017). It has been suggested that air respiration of the snakehead via the cough-like ventilation mechanism is water dependent (Ishimatsu and Itazawa, 1981), and therefore that the snakehead would be unable to survive in terrestrial habitats for very long (Liem, 1984). An alternative mechanism for the aerial survival of fish could be ammonia poisoning. In water, the ammonia products of fish are excreted easily and diffuse rapidly (Mommsen and Walsh, 1992; Wood, 1993). Out of water, however, the ammonia excretion of the fish may be blocked, which could lead to an accumulation of ammonia in the fish body (Chew et al., 2003; Sayer, 2005). Therefore, it can be hypothesized that the limited aerial survival of the northern snakehead could be attributed to excess ammonia accumulation.

We assumed that the aerial survival of the snakehead is involved not only with suprabranchial chamber respiration but also with physiological regulations, such as depressed metabolic demands, enhanced blood oxygen-carrying capacity and enhanced anaerobic metabolism. The oxygen consumption of fish with normal or injured suprabranchial chambers was compared to test the contribution of the suprabranchial chamber to oxygen uptake. VO_2_, blood parameters, lactate content of liver and muscle, and plasma ammonia were also assessed to test the changes in oxygen-carrying capacity, anaerobic metabolism and ammonia accumulation of the fish when switched from water-breathing to air-breathing states.

## RESULTS

**Fig. 1.**
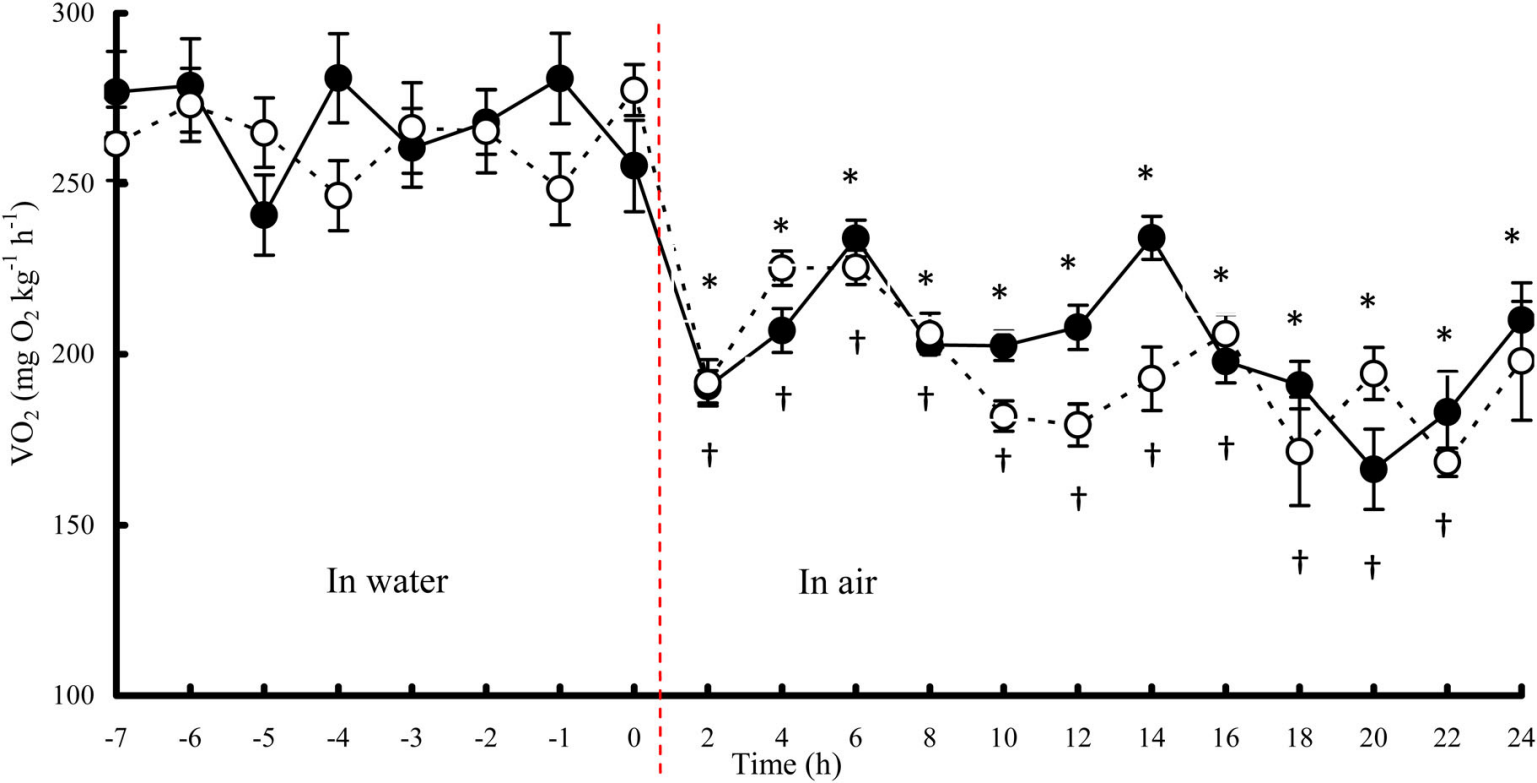
**Oxygen consumption rate (VO_2_) of northern snakeheads switched from water respiration to aerial respiration.** *n*=14 for each group. Data are presented as the mean±s.e.m. The values with symbols (*, control group; †, suprabranchial organ group) were different from the resting VO_2_ in water by a paired samples *t*-test (*P*<0.05). Filled circles with solid line, control group; open circles with dashed line, suprabranchial organ group.

The VO_2_ of both the control and suprabranchial organ-injured fish decreased when switching from water respiration to aerial respiration (Fig. 1). Resting VO_2_ in air (VO_2Air_) was significantly lower than resting VO_2_ in water (VO_2Water_), with decreases of 22.4% in the control and 23.5% in the injured fish (Table 1). Resting VO_2Air_ did not significantly differ between the control and injured fish. The aerial survival time of the injured fish was 19.0 h, nearly 4 h less than that of the control (22.9 h).

**Table 1.**
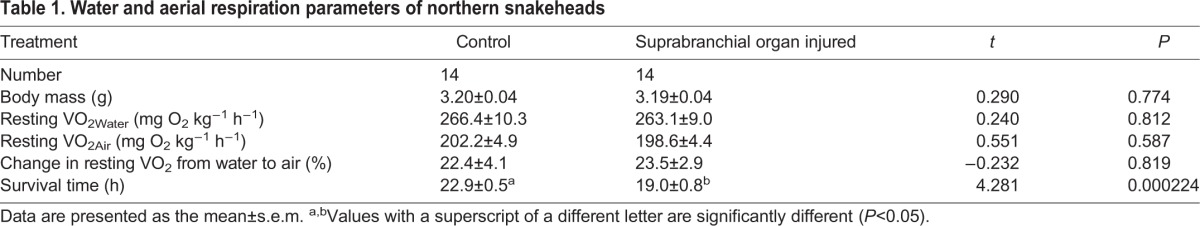
**Water and aerial respiration parameters of northern snakeheads**

**Fig. 2.**
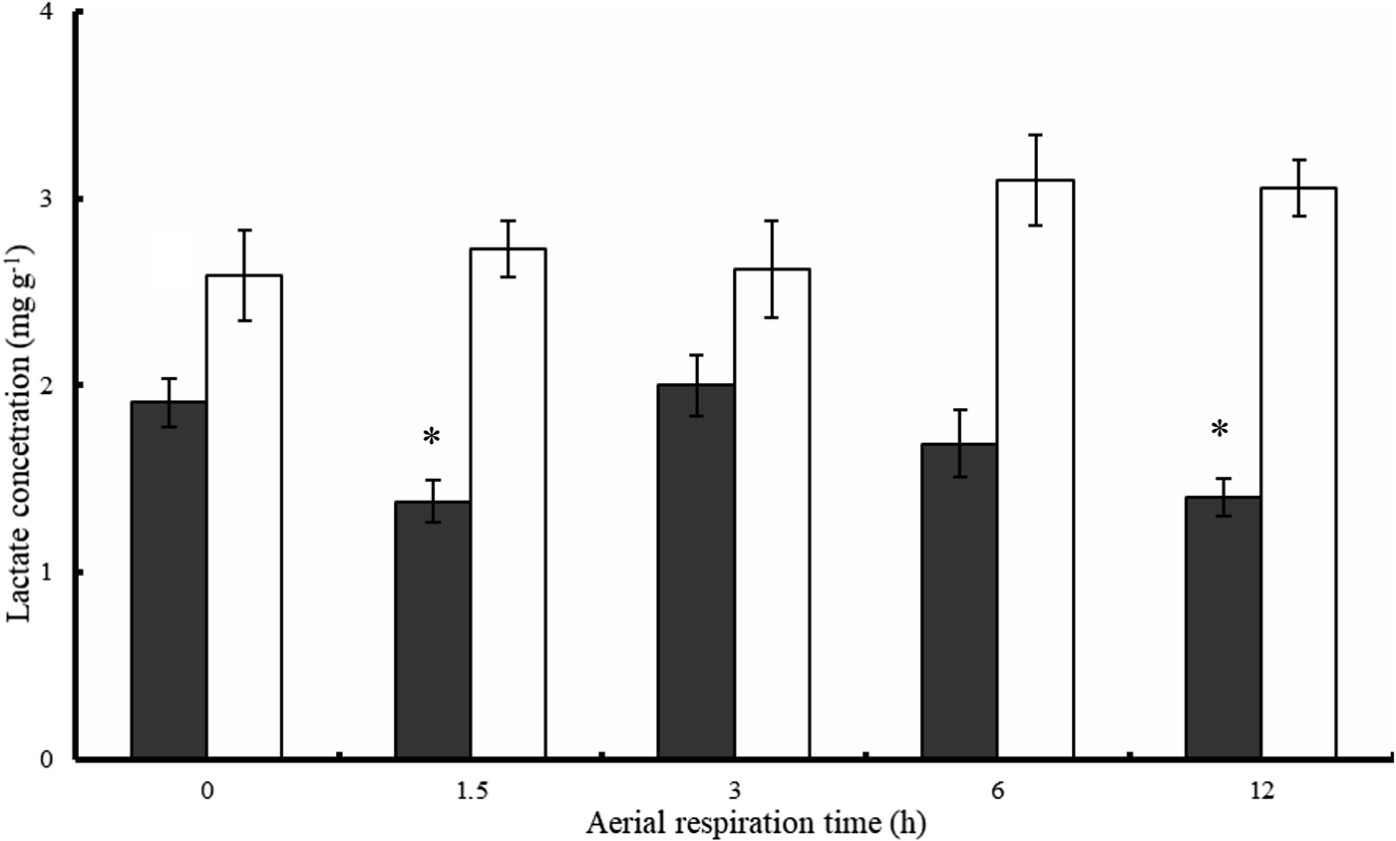
**Lactate concentrations in white muscle and liver of northern snakeheads switched from water respiration to aerial respiration.** *n*=10, 9, 10, 10 and 9 for the fish post-aerial respiration for 0 (in water), 1.5, 3, 6 and 12 h, respectively. Data are presented as the mean±s.e.m. The values of each organ with asterisks were different from values of the fish in water (0 h) by an independent samples *t*-test (*P*<0.05). Filled column, white muscle; open column, liver.

The oxygen-carrying capacity related parameters, including red blood cell (RBC) count (RBCC), hemoglobin concentration (Hb) and mean cellular hemoglobin content (MCH), of the fish did not change markedly when switching from water respiration to aerial respiration (Table 2). The RBC length (RBCL), width (RBCW) and area (RBCA) of the fish after several hours of aerial respiration were significantly higher than those of the fish after water respiration. The lactate content ranged from 2.6 to 3.1 mg g^−1^ in the liver and from 1.4 to 1.9 mg g^−1^ in the white muscle of the fish (Fig. 2). The liver lactate content did not significantly change, while the white muscle lactate content remained unchanged (at 3 or 6 h) or significantly decreased (at 1.5 and 12 h) when switching from water respiration to aerial respiration.

**Table 2.**
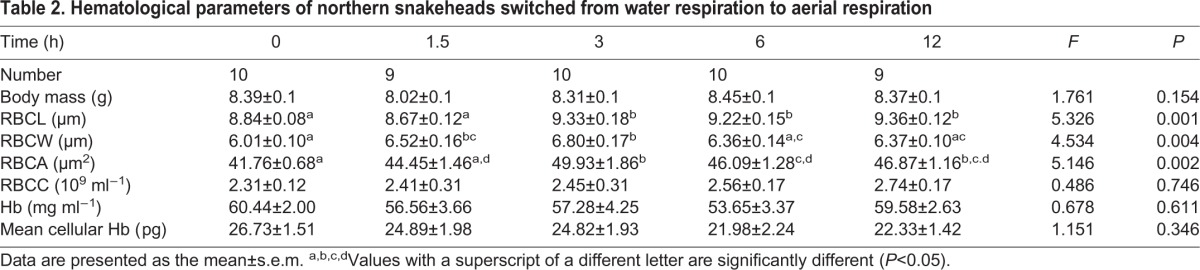
**Hematological parameters of northern snakeheads switched from water respiration to aerial respiration**

**Fig. 3.**
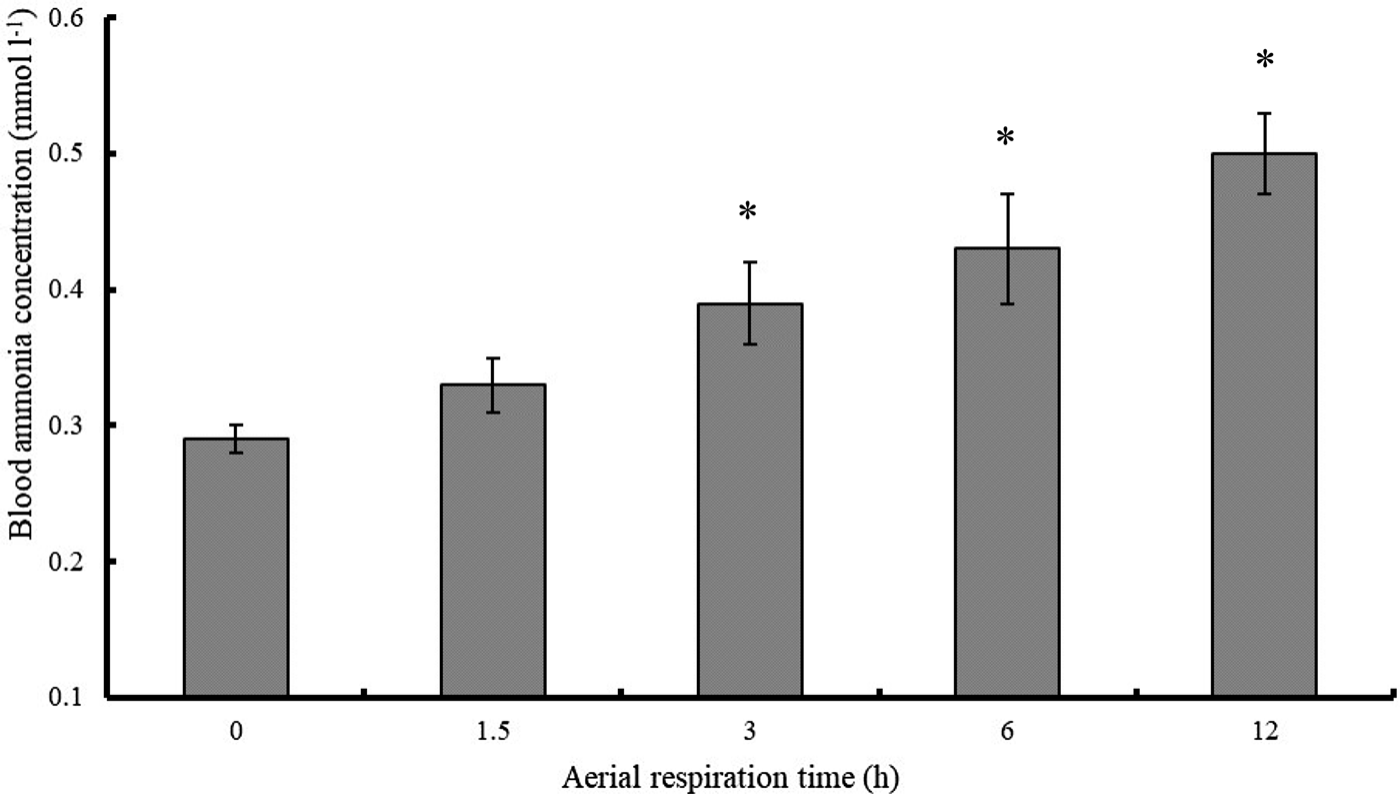
**Blood ammonia concentrations of northern snakeheads switched from water respiration to aerial respiration.** *n*=10, 9, 10, 10, and 9 for the fish post-aerial respiration for 0 (in water), 1.5, 3, 6 and 12 h, respectively. Data are presented as the mean±s.e.m. The values with asterisks were different from values of the fish in water (0 h) by an independent samples *t*-test (*P*<0.05).

The blood ammonia concentration of the fish in water was 0.29 mmol l^−1^ and tended to increase during aerial respiration (Fig. 3). After 12 h of aerial respiration, the blood ammonia concentration reached 0.50 mmol l^−1^.

## DISCUSSION

The suprabranchial chamber of the snakehead is an important accessory breathing organ that allows gas exchange in air (Ishimatsu and Itazawa, 1981; Glass et al., 1986). However, in our study, injury to the suprabranchial chamber did not change the VO_2Air_ of the northern snakehead, which suggests that organs other than the suprabranchial chamber also contribute to aerial respiration. Consistently, previous histological studies have shown that the roof of the buccopharynx and the surface of the tongue of the snakehead are also vascularized structures (Ishimatsu et al., 1979; Ishimatsu and Itazawa, 1983a,b).

Consistent with our previous study (Li et al., 2017), the snakeheads showed lower VO_2Air_ than VO_2Water_ (Fig. 1). A similarly reduced VO_2_ in air has also been observed in the Atlantic silverside, *Menidia menidia* (Garey, 1962; Martin et al., 2004). A reduced VO_2_ in fish can be attributed to a lower capacity for gas exchange or a downregulated metabolic demand. It has been proposed that the ventilation of the snakehead is inefficient during air exposure (Ishimatsu and Itazawa, 1981; Li et al., 2017), which limits its terrestrial survival capacity (Liem, 1984). However, the reduced VO_2_ of the snakehead in air might be due to different mechanisms, as the group with suprabranchial organ injuries maintained a VO_2_ equal to that of the fish with intact organs, suggesting that this species has sufficient oxygen exchange capacity. Therefore, the reduced VO_2_ of the snakehead in air can be ascribed to a downregulation of metabolic demand (Li et al., 2017). A lower metabolic rate is meaningful for fish in extreme environments, allowing them to save energy, to reduce end-product accumulation, and to survive for longer periods (Ginneken and Thillart, 2009).

RBCs and Hb are useful indicators of the blood oxygen-carrying capacity. Fish with a larger RBCC have greater blood oxygen-carrying capacity. For fish in water, the blood oxygen-carrying capacity may increase when faced with an oxygen shortage (Weber and Fago, 2004), and splenic erythrocyte release can increase RBCC and Hb (Randall, 1982; Murad et al., 1990; Pearson and Stevens, 1991). However, these hematological parameters of some air-breathing fish may not change during air exposure, as sufficient oxygen supply can be maintained by the air-breathing organ (Cruz et al., 2013). Similarly, in our study, the RBCC, Hb and MCH of the snakehead showed no marked changes in air compared to those in water, suggesting a sufficient oxygen supply. Interestingly, the RBC size increased when the fish were exposed to air, suggesting some extent of erythrocyte swelling, which can improve oxygen transport in fish (Wendelaar Bonga, 1997). Our results suggest that the aerial metabolic demand of snakeheads depends more on the regulation of RBC surface area than on the numbers of RBCs.

Another mechanism that allows fish to withstand environments with low oxygen availability is anaerobic metabolism (Hochachka et al., 1996). In our study, the anaerobic metabolism of the snakehead was not increased in air, as reflected by the unchanged liver lactate concentration and even decreased white muscle lactate concentration during aerial respiration (Fig. 2). This suggests that anaerobic metabolism has a minor contribution to the aerial survival of the northern snakehead. Unaffected liver and white muscle lactate has also been observed in another air-breathing fish, *Pterygoplichthys anisitsi* (Cruz et al., 2013).

Ammonia is the most toxic of the respiratory gases of fishes (Ip et al., 2001). When exposed to air, some fishes can maintain unchanged blood ammonia concentrations by reducing the rates of proteolysis and amino acid catabolism, by continuing to excrete ammonia, or by converting ammonia to less toxic forms, allowing them to survive in air for a long time; these species include the giant mudskipper (*Periophthalmodon schlosseri*), the swamp eel (*Monopterus cuchia*) and the African lungfish (*Protopterus annectens*) (Anderson, 2001; Ip et al., 2004; Chew et al., 2005). In contrast, accumulation of blood ammonia generally appears in fishes with reduced ammonia excretion (Chew et al., 2003), which may limit their ability to survive in air. Similarly, in our study, the blood ammonia of the northern snakehead accumulated during aerial respiration, which may partly explain its limited aerial survival time (23 h).

In conclusion, when northern snakeheads are exposed to air, their survival is positively associated with a combination of factors, including the respiration of suprabranchial chambers and other accessory respiratory organs, depressed metabolic demands and increased oxygen transport, but is negatively associated with the accumulation of blood ammonia. The regulation of anaerobic metabolism has a minor contribution to aerial survival of the northern snakehead.

## MATERIALS AND METHODS

The snakeheads were obtained from Huashan Hatchery, Guangdong Province, China. The fish were acclimated in a rearing system at the Fisheries Science Institute of Southwest University. The water temperature was 25±1°C, and the photoperiod was 12 h light: 12 h dark. The oxygen concentration was >6 mg l^−1^, and the ammonia concentration was <0.015 mg l^−1^. Animal handling and experiments were approved by School of Life Sciences, Southwest University (LS-SWU-1612) and were conducted according to the requirements of Environment and Housing Facilities for Laboratory Animals of China (GB/T14925-2001).

To test the change in VO_2_ due to suprabranchial organ injury, the snakeheads were divided into two groups: a control group (*n*=14) and an injured group (*n*=14). The fish were fasted for 24 h, and the body mass was weighed (∼3 g each). Then, the fish were individually transferred into respiratory chambers and adapted overnight before determination of VO_2Water_ and VO_2Air_. The structure of the respiratory chamber and the methods for VO_2_ determination were described by Li et al. (2017). Briefly, the chamber (30 ml) can be switched from a flow-through water phase to a closed air phase by regulating the inlet and outlet valves. For water respiration, the dissolved oxygen concentration (mg O_2_ L^−1^) was measured at the outlet by a fiber optic sensor system (Mircrox TX3, PresSens Precision Sensing GmbH, Regensburg, Germany), and the water flow rate (L h^−1^) was measured by collecting water over a given time. The VO_2Water_ (mg O_2_ h^−1^) was calculated by multiplying the water flow rate by the difference in dissolved oxygen concentration between the fish chamber and the control chamber. The dissolved oxygen in the outlet water was maintained at >70% saturation concentration to avoid causing hypoxic stress. For VO_2Air_ determination, the water in the chamber was discharged, and a near-saturated humidity condition was maintained in the chamber. VO_2Air_ was determined in an intermittent flow pattern. The initial oxygen partial pressure of the air was measured, and the chamber was sealed for 90 min; then, the final oxygen level was measured. The change in air volume due to air breathing was determined from the reading on a 1-ml syringe connected to the inlet. Atmospheric pressure was recorded during each measurement. Then, the air in the chamber was refreshed for 30 min, and the next determination loop was started. The oxygen concentration in the air (mg O_2_ l^−1^) was obtained from the values of oxygen partial pressure, temperature and atmospheric pressure. VO_2Air_ (mg O_2_ h^−1^) was calculated as *VO*_2*Air*_=(*O*_2*i*_×*V*_*i*_−*O*_2*f*_×*V*_*f*_)/*t*, where O_2i_ and O_2f_ are the initial and final oxygen concentrations of the air (mg O_2_ l^−1^), respectively; V_i_ and V_f_ are the initial and final air volume, respectively; and t is the breathing time (h). The volume of the fish body was estimated by assuming a body density of 1 kg l^−1^. One chamber without fish was set as a control for either water or air respiration. A mass-specific value of VO_2_ was obtained by dividing by body mass (kg). VO_2Water_ was measured for both groups at 1-h intervals for 8 h, and the mean value was taken as the resting VO_2Water_. For the injured group, we sheared the ventral and posterior walls of the suprabranchial chamber using surgical scissors. After 30 min of adaptation, the VO_2Air_ of the two groups was determined at 2-h intervals for 24 h. The mean values were taken as the resting VO_2Air_.

Another series of experiments was conducted to test the changes in hematological and biochemical parameters during aerial exposure. Snakeheads (∼8 g) were individually placed in moist beakers (1 l) in the air at 25±1°C. After 0 (control), 1.5, 3, 6 or 12 h of aerial exposure, fish were anaesthetized by immersion in MS222 solution (tricaine methanesulfonate) for 2-3 min (Luo et al., 2013). A blood sample was taken by syringe from the caudal vein and divided into four parts to determine the RBCC, RBC size, Hb and blood ammonia concentration. The liver and a piece of white muscle below the dorsal fin were sampled on ice using surgical scissors for the determination of lactate concentration. The RBCC was determined with a Neubarner counter, and the RBCL and RBCW of 50 randomly selected RBCs were determined under a digital light microscope (EV5680, Aigo Company, Beijing, China) linked to a computer. The RBCA was calculated using the formula: RBCA=RBCL×RBCW×π/4 (Gao et al., 2007). Hb was determined by the alkalinized hemoglobin method (Dacie and Lewis, 1984). The MCH was calculated by the ratio of Hb to RBCC. The blood ammonia concentration was determined with a blood ammonia determination kit (Jiancheng Biotech Co., Ltd, Nanjing, China). White muscle and liver were each mechanically homogenized and diluted 10-fold with ice-cold saline solution (0.65%), and the contents of lactate were measured using a lactic acid assay kit (Jiancheng Biotech Co., Ltd, Nanjing, China).

### Data analysis

Data were calculated using Microsoft Excel 2007 and statistical analyses were performed using IBM SPSS 11.5. The independent samples *t*-test was used to compare resting VO_2_, change in VO_2_ when switching from water to air, and survival time between the control and injured groups. The paired samples *t*-test was used to compare the resting VO_2Water_ with the VO_2Air_ at each time point. One-way ANOVA followed by a multiple comparisons test (LSD) was used to test the changes in the blood parameters along time points during aerial exposure. The independent samples *t*-test was used to compare lactate content and ammonia concentration at each time point during aerial respiration with those during water respiration. Data are presented as the mean±s.e.m. Differences were considered statistically significant when *P*<0.05.
